# Ultrathin Al–air batteries by reducing the thickness of solid electrolyte using aerosol jet printing

**DOI:** 10.1038/s41598-022-14080-6

**Published:** 2022-06-13

**Authors:** Yuxin Zuo, Ying Yu, Junyan Feng, Chuncheng Zuo

**Affiliations:** 1 Jiaxing Nanhu University, Jiaxing, 314000 China; 2 College of Information Science and Engineering, Jiaxing University, Jiaxing, China

**Keywords:** Energy storage, Fuel cells

## Abstract

Flexible Al–air batteries have great potential in the field of wearable electronic devices. However, how to reduce the thickness of the battery and improve their applicability in wearable applications is still an unresolved thorny problem. Therefore, this article focuses on the strategies to minimize the thickness of the solid electrolyte for flexible Al–air batteries. In this paper, an innovative aerosol jet printing method is used to prepare the ultrathin neutral electrolyte with a thickness of 18.3–74.5 μm. This study discusses the influence of the thickness and ion concentration on the conductance of the electrolyte in detail. The ultrathin electrolyte has been applied to the flexible Al–air battery, and the battery performance has been explored. The cell pack composed of single cells is light and thin, and can successfully drive small electrical equipment. This study provided new ideas for the preparation of ultrathin electrolyte for flexible energy products.

## Introduction

Al–air batteries have received extensive attention from scientific researchers due to their light weight, high energy density, and environmental friendliness^1,2^, and have been initially applied in the fields of biosensors, folding screens, flexible circuit boards and etc.^3,4^ However, the thickness of the current Al–air battery is difficult to match the requirements of wearable electronic devices, which would affects the portability and comfort of the product. Therefore, the preparation of ultrathin Al–air batteries is particularly important. In fact, the thicknesses of the anode and the cathode are difficult to further decrease, while the solid electrolyte has more space for optimization.

At present, the solid electrolytes of Al–air batteries are mainly prepared by sol-gel^3,5^, polymerization, casting^6,7^ and inkjet printing^8^, and the thickness is generally between several millimeters to several centimeters^9,10^. In related research on other types of batteries, we noticed that in order to improve the electrochemical performance and portability of lithium-ion batteries, scientists tried to prepare ultrathin solid-state gel electrolytes. Deiner et al.^11^ have successfully prepared ultrathin gel electrolytes for lithium-ion batteries based on aerosol jet printing. The thickness of the electrolyte is about 100 microns, and thanks to its thickness advantage, higher ionic conductance is obtained compared to traditional solid-state gel electrolytes. Previous studies have also shown that the ion diffusion time in the electrolyte is proportional to the square of the electrolyte thickness^9^, and the conductance of the electrolyte is inversely proportional to the electrolyte thickness^12^. It can be seen that reducing the thickness of the electrolyte can not only improve the portability of the battery, but also help to improve the conductance of the electrolyte, thereby improving the electrochemical performance of the battery.

Existing researches on electrolytes of Al–air batteries mainly focus on how to improve the conductivity of the solid electrolyte, and pay less attention to the thickness^13^. In fact, the conductance of the electrolyte is directly related to the thickness, and reducing the thickness could increase the conductance. According to relevant literatures, the thickness of the solid electrolyte in the existing research for Al–air batteries is generally several millimeters or even several centimeters^14,15^. Conventional preparation methods are difficult to fabricate micron-scale electrolytes, and the controllability of the thickness is unsatisfactory. Currently, there are few studies on the preparation and characterization of micron-scale electrolytes.Therefore, according to the above-mentioned idea of ultrathin electrolytes for lithium-ion batteries^11^, this paper will try to prepare ultrathin electrolytes for Al–air batteries by aerosol jet printing. Aerosol jet printing is a novel method that can prepare ultrathin functional membrane, and can precisely control the thickness of the membrane^16^. Currently, it has been applied in preparation of transistors^17^, photovoltaics^18^, sensors^19^ and etc.

In this paper, an ultrathin electrolyte was prepared by aerosol printing, using polyvinyl alcohol (PVA) as the gelling agent and NaCl as the salt source. The ultrathin electrolyte was assembled in a flexible Al–air batteries with carbon cloth as the air cathode and aluminum foil as anode. Before battery testing, the temperature dependency of the ions conductivity, and the effects of thickness and concentration on the conductance of the electrolyte were explored. Next, the power density, impedance, discharge stabality and capacity of the Al–air batteries were tested with the electrolytes of different thicknesses and concentrations. Finally, a flexible cell pack was assembled for practical application. We hope that this study would provide a novel method for the preparation of ultrathin gel electrolytes and enlarge the application of flexible Al–air batteries.

## Results and discussion

### Characterization of the ultrathin electrolyte

**Figure 1:**
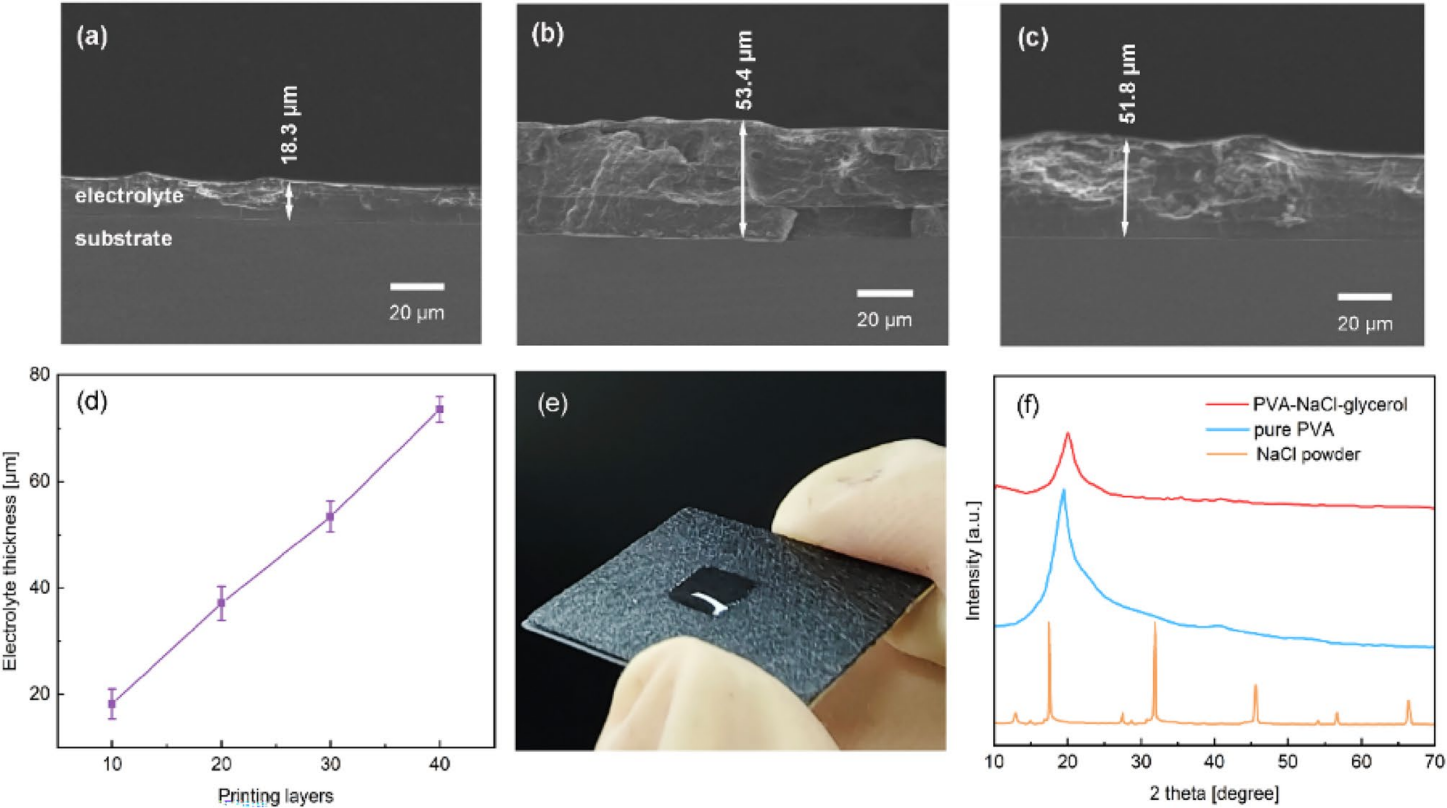
(**a**) Cross-sectional SEM of the electrolyte printed10 layers, (**b**) printed 30 layers non-continuously and (**c**) continuously. (**d**) The thickness of the electrolyte depends on the number of printing layers. (**e**) The photo of the electrolyte prepared by aerosol jet printing. (**f**) XRD patterns of NaCl, PVA and PVA-NaCl-glycerol.

Aerosol jet printing is to atomize the electrolyte ink and deposit it on the substrate. One of the difficulties in achieving high-presicion and controllable printing lies in the preparation of electrolyte ink^16^. It is required that the electrolyte ink could be atomized during the printing process, and then could be solidified to form a gel layer after being left to dry. Figure 1a is the cross-sectional scanning electron microscope (SEM) image of the solid electrolyte printed with 10 layers, and the thickness of the electrolyte is about 18.3 μm. It can be seen from the image that there is almost no gap at the electrode/electrolyte interface. Aerosol jet printing does achieve seamless and conformal deposition of electrolyte on the substrate. After measurement, the electrolyte thicknesses of printed 20, 30 and 40 layers are 37.8, 51.8 and 74.5 μm. In order to further verify the controllability of the thickness, a non-continuous segmented printing was conducted. That is, after printing 10 layers, the drying process is performed immediately, and then the printing is continued on the dried electrolyte. Figure 1b,c are the cross-sectional views of the solid electrolyte printed 30 layers non-continuously and continuously. In non-continuous segmented printing, the thickness of each 10 layers is almost the same, and the total thickness is very close to that of continuous printing 30 layers. Figure 1d shows the relationship between electrolyte thickness and printing layers. The thickness of the electrolyte has an approximately linear relationship with the printing layers, which fully confirms the high precision and controllability of the preparation of the electrolyte in this study. The printed electrolyte is shown in Fig. 1e.

Figure 1f shows the X-ray diffraction (XRD) patterns of the PVA-NaCl-glycerol electrolyte. It can be seen from the figure that the diffraction peaks of pure PVA and PVA-NaCl-glycerol both appear at the position of 2θ = 19.6°. Compared with pure PVA, the diffraction peaks of PVA-NaCl-glycerol are significantly reduced, and the degree of dispersion is increased. The reason is that both water and glycerol are solvents that could fully dissolve NaCl and prevent it from crystallizing effectively. Therefore, the addition of glycerol and NaCl will not change the crystal structure of the polymeric matrix. A similar conclusion was also obtained in the researches of Li^20^. and Liu et al.^21^. The XRD patterns also confirmed the homogeneity of the PVA-NaCl-glycerol electrolyte.

### Electrochemical performance of the ultrathin electrolyte

**Figure 2:**
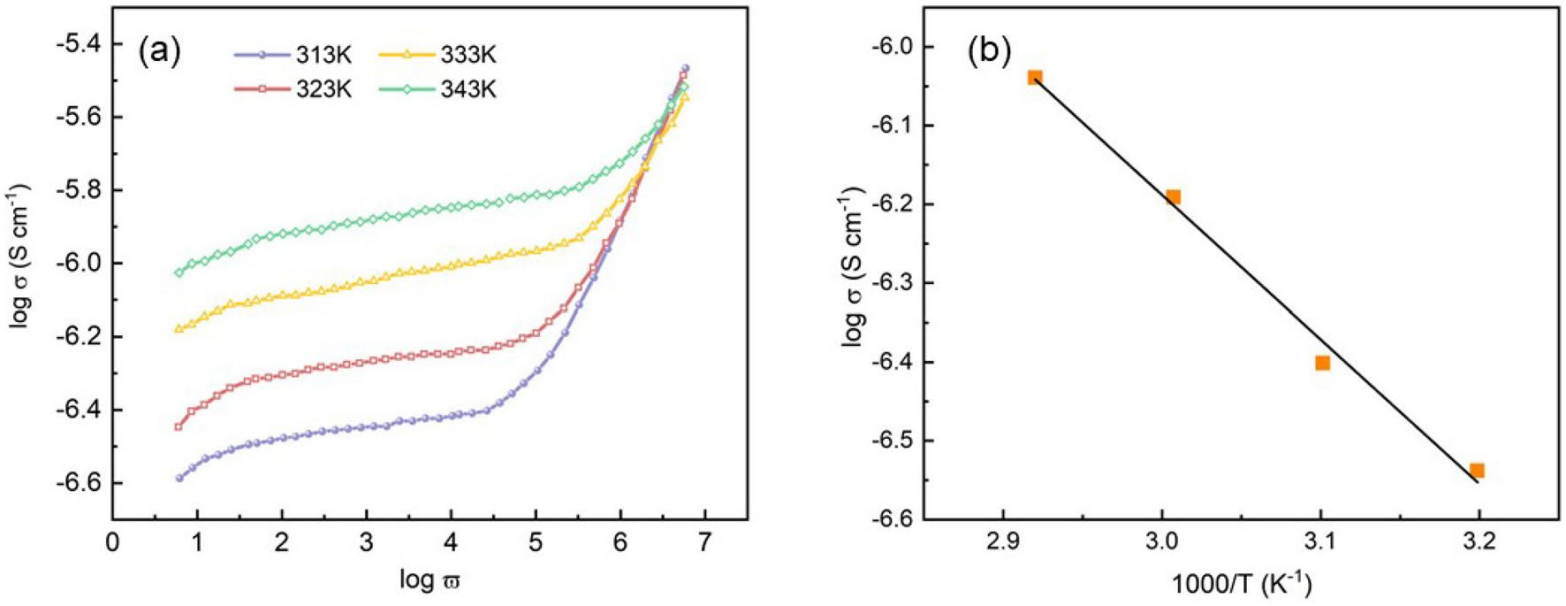
(**a**) Frequency dependent conductivity of the solid electrolyte, (**b**) log $\sigma$ versus 1000/T plot of the electrolyte. The solid line indicates the linear fit.

The nature of ion transport in solid electrolytes can be analyzed by frequency-dependent electrical responses. This study will analyze impedance data for a temperature range of 303–343 K and a frequency range of 1–1 MHz. The conductivity σ could be expressed as the sum of dc and ac components according to Jonscher’s universal power law equation ^22^ as

1 $${\varvec{\sigma}} = {\varvec{\sigma}}_{{{\varvec{dc}}}} + \user2{A\omega }^{{\varvec{n}}}$$

where ${\varvec{\sigma}}_{{{\varvec{dc}}}}$ and $\user2{A\omega }^{{\varvec{n}}}$ are the frequency independent conductivity at low and high frequencies, respectively. $\user2{A }$ is a frequency independent and temperature dependent parameter, and ${\varvec{\omega}} = 2\user2{\pi f}$ is the angular frequency. ${\varvec{n}}$ is the temperature dependent frequency exponent which represents frequency dependent conductivity. Figure 2a shows the frequency dependence of conductivity at different temperatures of the solid electrolyte with a thickness of 74.5 μm and a concentration of 1.8 mol. It can be seen from the figure that frequency independent conductivity appears in the low frequency region, which would correspond to the typical dc conductivity. The dc conductivity values corresponding to all temperatures were measured by extrapolating the low frequency plateau regions to the log ${\varvec{\sigma}}$ axis and obtained from their intercepts at zero frequency. The ionic conductivity increases with the increasing frequency in the high frequency region, which can be attributed to the forward and backward ion displacements occurring simultaneously thus facilitating the frequency dependence of ionic motion in accordance with Jonscher’s universal power law behavior. As the temperature increases, the frequency at which the dispersion becomes prominent shifts to higher frequency region, which means the bulk relaxation shifts to higher frequencies.

The dc conductivity values of the electrolytes at various temperatures are plotted against inverse of temperature in Fig. 2b. The linear variation of log ${\varvec{\sigma}}$ versus 1000/T could be explained by the Arrhenius-type thermally activated process

2 $${\varvec{\sigma}}_{{{\varvec{dc}}}} = {\varvec{\sigma}}_{{\varvec{o}}} {\mathbf{exp}}\left( { - {\varvec{E}}_{{\varvec{a}}} /{\varvec{kT}}} \right)$$

where ${\varvec{\sigma}}_{{\varvec{o}}}$ is the pre-exponential factor, ${\varvec{E}}_{{\varvec{a}}}$ is the activation energy, ${\varvec{k}}$ is the Boltzmann constant, and ${\varvec{T}}$ is the absolute temperature. The observed log ${\varvec{\sigma}}$ versus 1000/T plot in the figure is consistent with the theory established by Tareev et al.^23^. The activation energy of the electrolyte is calculated as about 0.38 eV, indicating a lower ions’ hopping barrier. In the study of Druger et al.^24^, the change of conductivity with temperature was attributed to the segmental (i.e. polymer chains) motion. When the temperature increased, the free volume of the system increased accordingly. The free volume around the polymer chain induces the motion of surrounding ions, therefore, an increase in the segmental motion of the polymer chains will cause an increase in conductivity.

In this study, in order to find the optical ultrathin electrolyte for Al–air battery, we have studied the influence of the electrolyte thickness and NaCl content on the electrochemical performance of the electrolyte. Conductance is an important parameter of the electrolyte, which will directly affect the performance of the batteries. The conductivity $\sigma$ of the ultrathin electrolyte was obtained via electrochemical impedance spectroscopy (EIS), using Pt / solid electrolyte / Pt symmetric cells.

3 $$\sigma = \frac{l}{RA}$$

where $l$ and $R$ are the thickness and bulk resistance of the solid electrolyte, and $A$ is the contact area. The ionic conductance of the electrolyte, which takes the thickness of the electrolyte into consideration^25^,

4 $$G = \sigma A/l$$

**Figure 3:**
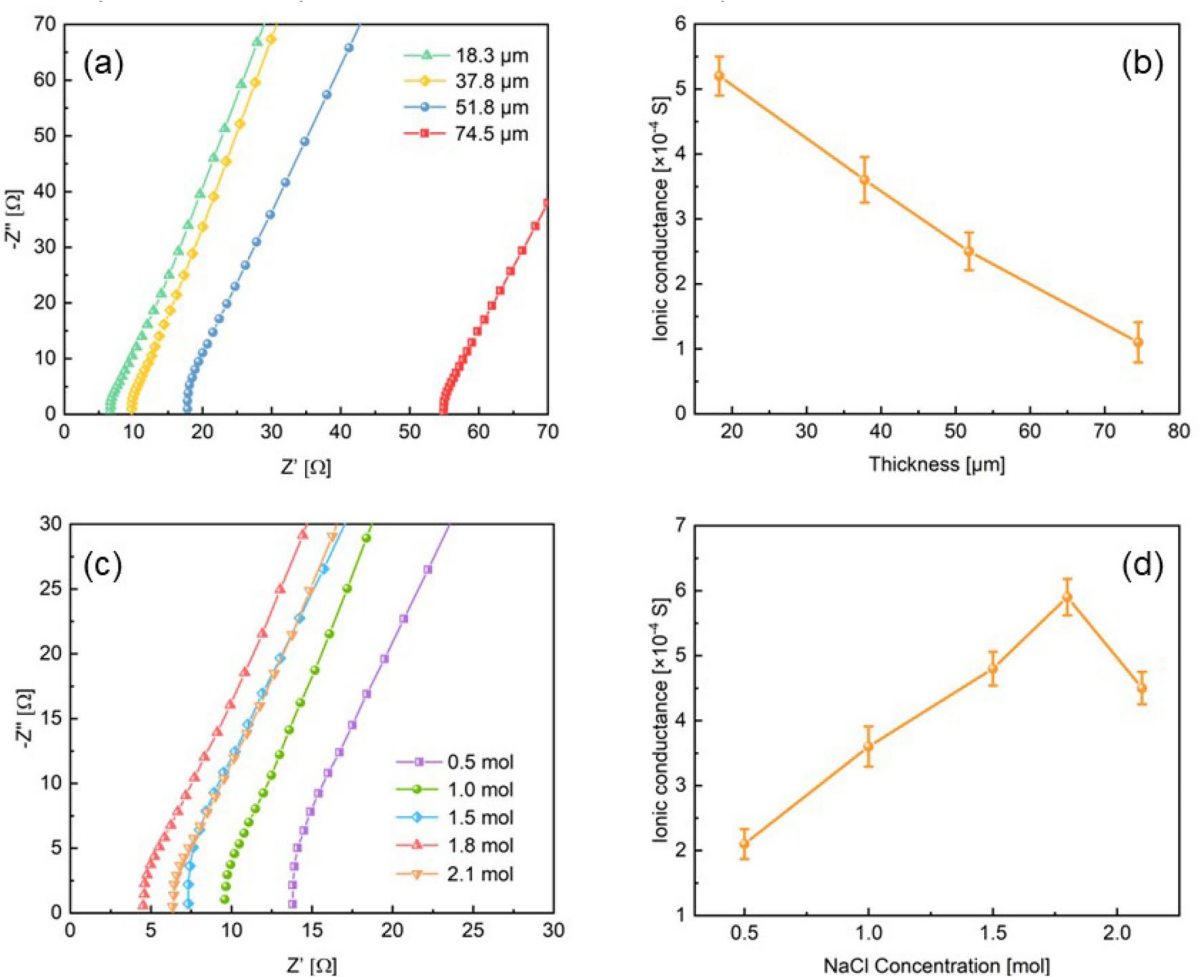
(**a**) EIS curves of the electrolyte with different thicknesses. (**b**) The thickness-dependent ionic conductance of the electrolyte. (**c**) EIS curves of the electrolyte with different NaCl content. (**d**) The NaCl content-dependent ionic conductance of the electrolyte.

Figure 3a shows the Nyquist plots of the solid electrolyte with different thicknesses when the NaCl concentration is 1.0 mol. From the intercept of the curves with real axis, it is apparent that as the electrolyte thickness increases, the impedance increases significantly. The conductance of the electrolytes with thicknesses of 18.3, 37.8, 51.8 and 74.5 μm are 5.2 × 10^–4^, 3.6 × 10^–4^, 2.5 × 10^–4^ and 1.1 × 10^–4^ S, respectively. The thickness-dependent ionic conductance of the electrolytes can be seen in Fig. 3b. Since the thickness of the electrolyte is thicker, the path of ion transport is farther, and the diffusion time is longer. The conductance of the electrolyte decreases as the thickness increases apparently. It can be seen that reducing the thickness of the electrolyte can effectively increase the conductance. Figure 3c is the Nyquist plots of 37.8 μm thick electrolytes with different NaCl content. It is observed that the NaCl content increase from 0.5 to 1.8 mol, and the conductance increases from 2.1 × 10^–4^ to 5.9 × 10^–4^ S. When the NaCl concentration continues to increase to 2.1 mol, the conductance decreases instead. The relationship between the conductance and NaCl content is shown in Fig. 3d. Excess NaCl beyond the peak concentration will inhibit the conductance of the electrolyte because the increase in electrolyte viscosity limits the ions mobility^26,27^.

### Performance of the Al–air battery with ultrathin electrolyte

**Figure 4:**
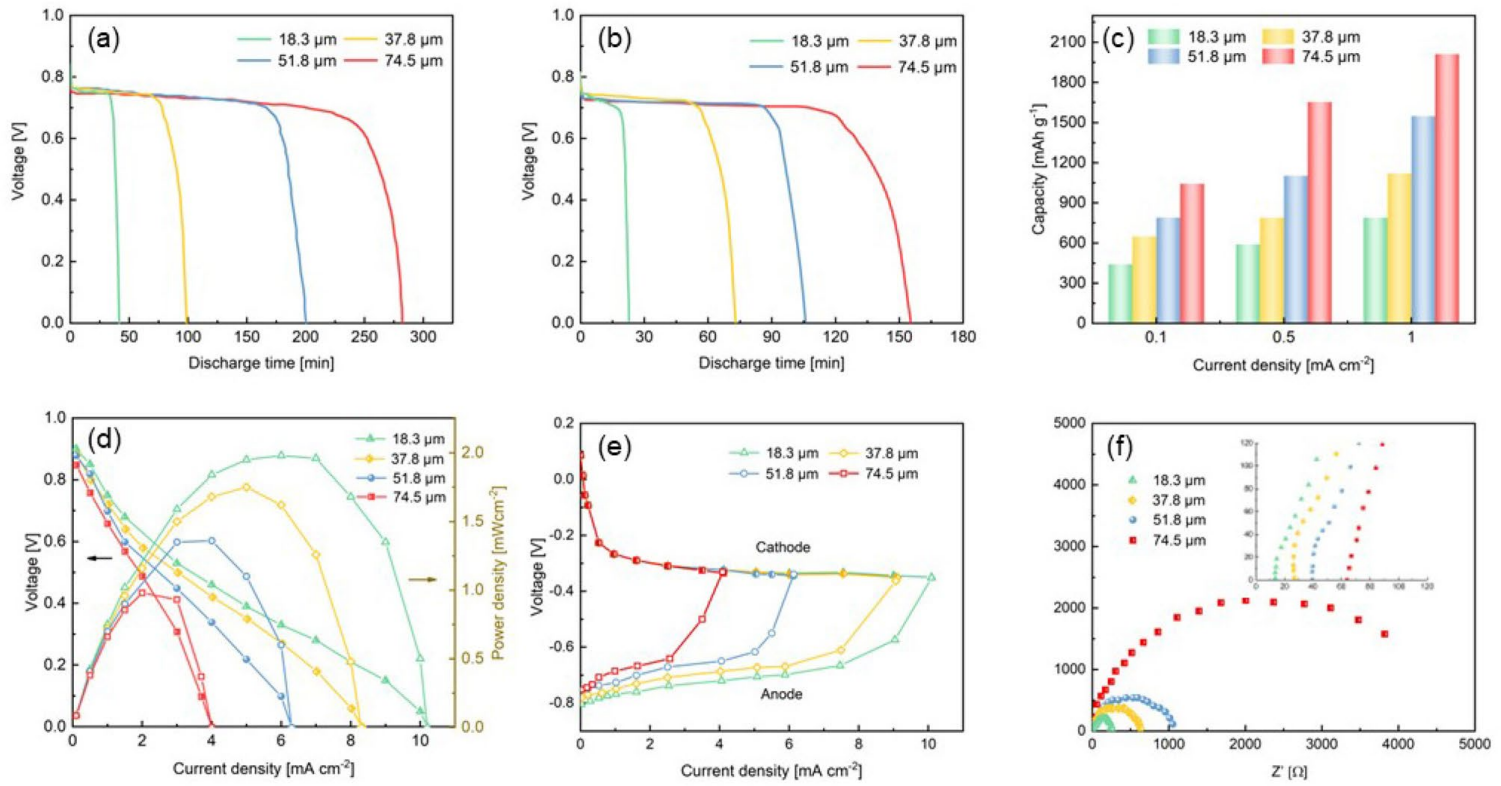
The discharge profiles of the Al–air battery at (**a**) 0.1 and (**b**) 0.5 mA cm^−2^. (**c**) Specific capacity of the Al–air batteries with electrolytes of different thicknesses. (**d**) The polarization curves and power-density profiles of the Al–air batteries. (**e**) Single electrode polarization. (**f**) EIS analysis of the Al–air battery.

In order to further investigate the electrochemical performance of the ultrathin electrolyte, we apply it to the flexible Al–air battery to test its applicability. When the NaCl content in the electrolyte is 1.0 mol, Fig. 4a,b present the discharge behavior of the Al–air batteries using 18.3, 37.8, 51.8 and 74.5 μm electrolyte at a constant current density of 0.1 and 0.5 mA cm^-2^. The open-circuit voltage (OCP) of the Al–air battery with the 18.3 μm electrolyte is higher than the others, which is consistent with the results of the ionic conductance. The discharge voltage plateaus of the Al–air batteries are about 0.78 V and 0.75 V at 0.1 and 0.5 mA cm^-2^. Compared with Al–air batteries using alkaline solid electrolyte^28,29^, the flat plateau of batteries using neutral gel electrolyte is lower. One reason is that the oxide layer (Al_2_O_3_) on the surface of Al anode is difficult to remove under neutral electrolyte conditions^30^. Another reason lies in the lower reaction kinetics of Al oxidation in saline environment. However, compared to the neutral gel electrolyte-based Al–air batteries in other studies, the discharge plateau voltage of this study has obvious advantages^14^. It can be seen from the galvanostatic discharge curve that the discharge time is shortened with the decrease of the electrolyte thickness, and the similar conclusions have been obtained in the study of Wang^31^. In a thinner electrolyte, the content of Cl^-^ ions is limited. On the surface of the Al anode, the reacted aluminum will take off the Al_2_O_3_ shell, and the shell will accumulate at the interface between the electrode and the electrolyte during the electrochemical reaction, which will increase the internal resistance of the battery and hinder the passage of ions. In addition, Cl^-^ ions in the thicker electrolyte are sufficient to reach the surface of Al anode and participate in the electrochemical reaction. Figure 4c shows the specific capacity of Al–air battery with the ultrathin electrolyte of different thicknesses. The specific capacity of the battery increases as the thickness increases of the electrolyte. When the electrolyte thickness is 74.5 μm, the specific capacity could reach 2011 mAh g^−1^ at the current density of 1 mA cm^-2^. Under the same discharge current density, the specific capacity obtained in this study is higher than that of the traditional Al–air battery with alkaline electrolyte^32,33^. This is mainly due to the serious self-corrosion of the Al anode under the condition of alkaline electrolyte. From the perspective of anode utilization, Al electrode under neutral electrolyte conditions are mainly mechanical loss rather than chemical loss^30^.

Figure 4d comparing the battery performance with different thicknesses of electrolyte. The increase in electrolyte thickness seriously affects the performance of the battery. The thickness of the electrolyte increases from 18.3 to 74.5 μm, the peak power density decreases from 1.98 to 0.98 mW cm^-2^, and the maximum current density decreases from 10.2 to 4 mA cm^-2^. Wang et al^31^. prepared a 2 mm thick ethanol gel electrolyte and applied it to Al–air battery, and the obtained power density of the battery is very close to the result of this study. Although the power density of the battery using this ultrathin NaCl electrolyte is lower than that with traditional liquid alkaline electrolytes^34,35^, it is sufficient for wearable flexible electronic devices such as medical diagnostic assays, biosensors and RFID tags, which only need to consume milliwatts or microwatts of power^36–38^. As can be seen in Fig. 4e, the cathode performance kept identical and straight, while there is a significant difference and mass transport loss at the anode side as the electrolyte thickness increases. Figure 4f shows that the battery ohmic resistance is greatly reduced from 63 Ω for 74.5 μm electrolyte to 12 Ω for 18.3 μm electrolyte. In the research of Yang et al.^39,40^, it has also been confirmed that the performance of the battery is affected by the thickness of the electrolyte. As the distance between the two electrodes decreases, the ion diffusion efficiency in the electrolyte increases, the internal resistance of the battery decreases accordingly, and the battery performance improves. Combining the specific capacity of the anode and the battery performance, it can be seen that for the ultrathin electrolyte, the thickness reduction of the electrolyte can effectively improve the performance of the Al–air battery, but at the expense of the specific capacity of the aluminum electrode.

**Figure 5:**
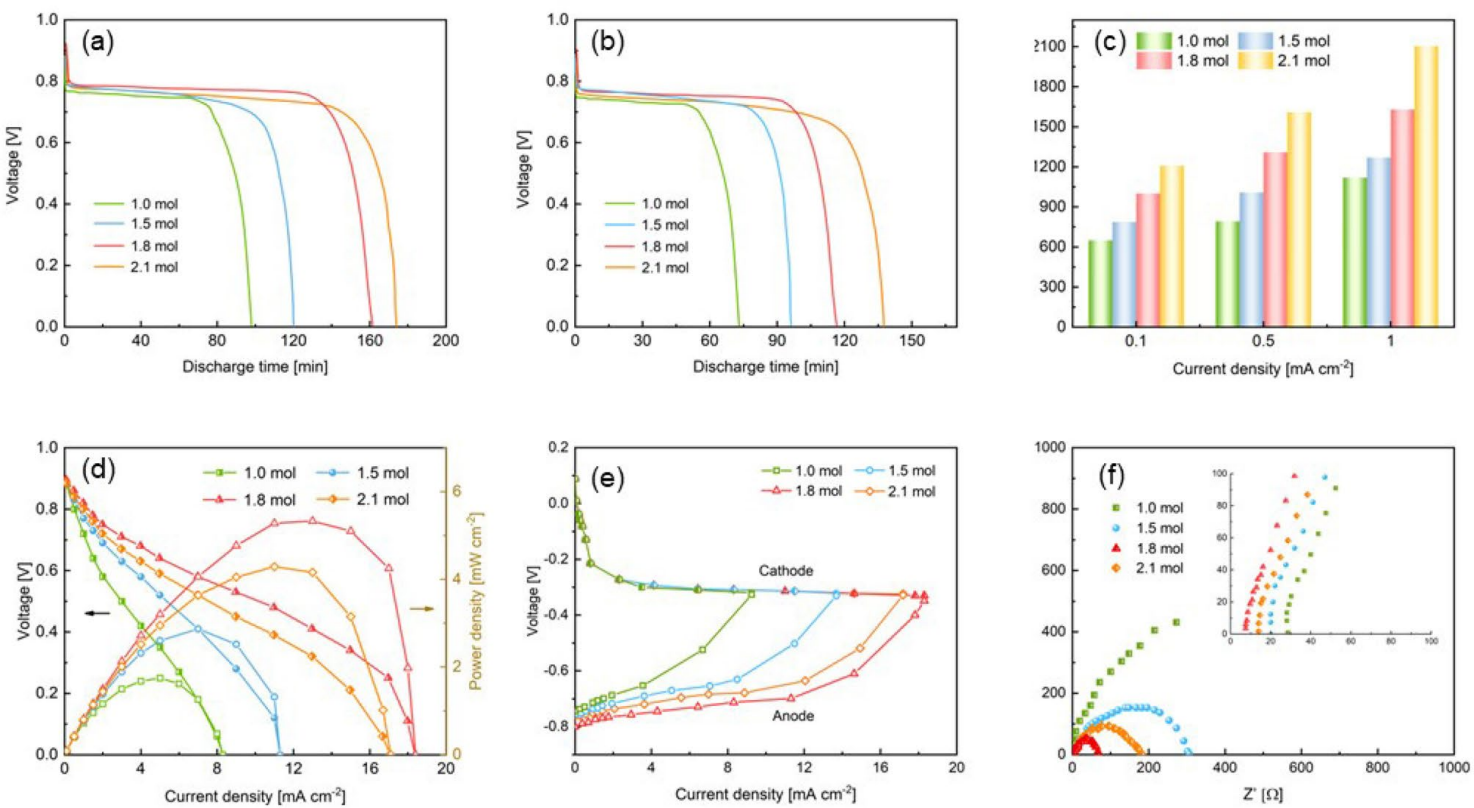
The discharge profiles of the Al–air battery at (**a**) 0.1 and (**b**) 0.5 mA cm^−2^. (**c**) Specific capacity of the Al–air batteries with electrolytes of different NaCl content. (**d**) The polarization curves and power-density profiles of the Al–air batteries. (**e**) Single electrode polarization. (**f**) EIS analysis of the Al–air battery.

In addition to the thickness, the concentration of Cl^-^ ions in the ultrathin electrolyte is also an important factor. Taking a 37.8 μm thick solid electrolyte as an example, Fig. 5a,b show the discharge curves of Al–air batteries with ultrathin electrolytes of different NaCl content at the current density of 0.1 and 0.5 mA cm^-2^. It can be seen from the figure that as the NaCl content increases, the discharge platform voltage increases, the stability is better, and the discharge time is prolonged. Figure 5c shows the relationship between the battery specific capacity and the concentration of NaCl in electrolyte. Increasing the NaCl content can significantly increase the specific capacity of the battery. A battery with an electrolyte containing 2.1 mol NaCl could obtain a specific capacity of 2105 mAh g^-1^, which is comparable to an Al–air battery with a 1 mm thick ethanol gel electrolyte^31^, and higher than a magnesium-air battery of similar electrolyte composition^41^. This may be due to the fact that, compared with traditional preparation methods, the distribution of the components in the electrolyte prepared by aerosol jet printing is extremely uniform, and the passage of Cl^-^ ions is more convenient. As shown in Fig. 5d, the battery performance is gradually improved as the NaCl content increased. When the NaCl content increases to 1.8 mol, the power density of the battery reaches 5.33 mWcm^-2^. Continue to increase the NaCl content, the performance of the battery will decrease instead. This is consistent with the influence of NaCl content on conductivity as shown in Fig. 3d. It can be explained that the content of NaCl that can be dissolved in the aqueous solution is limited. Similar conclusions have also appeared in the study of Wang et al.^30^. In Fig. 5e, it can be found that the major overpotential loss is from the anode side, where the aluminum oxidation reaction was greatly impeded due to the lack of hydroxyl ions. Although it is difficult to remove the A_l2_O_3_ protective film on the surface of the aluminum electrode in neutral electrolyte, Cl^-^ in the electrolyte can still attack the defects of the Al_2_O_3_ layer, exposing fresh Al to the electrolyte. Also, the cathode can also generate OH- for the anode reaction^42^. Therefore, with the increase of NaCl concentration, the anode performance can be improved. However, further increment of the NaCl concentration to 2.1 mol has no discernable effect on the anode side, which is probably because of the OH^−^ supply restriction from the cathode side. Figure 5f shows that higher NaCl concentration brings a large decrease in charge transport resistance as well as a reduced in ohmic resistance. Therefore, it can be concluded that an appropriate increase in the concentration of NaCl is beneficial to improve the battery performance for ultrathin electrolytes.

**Figure 6:**
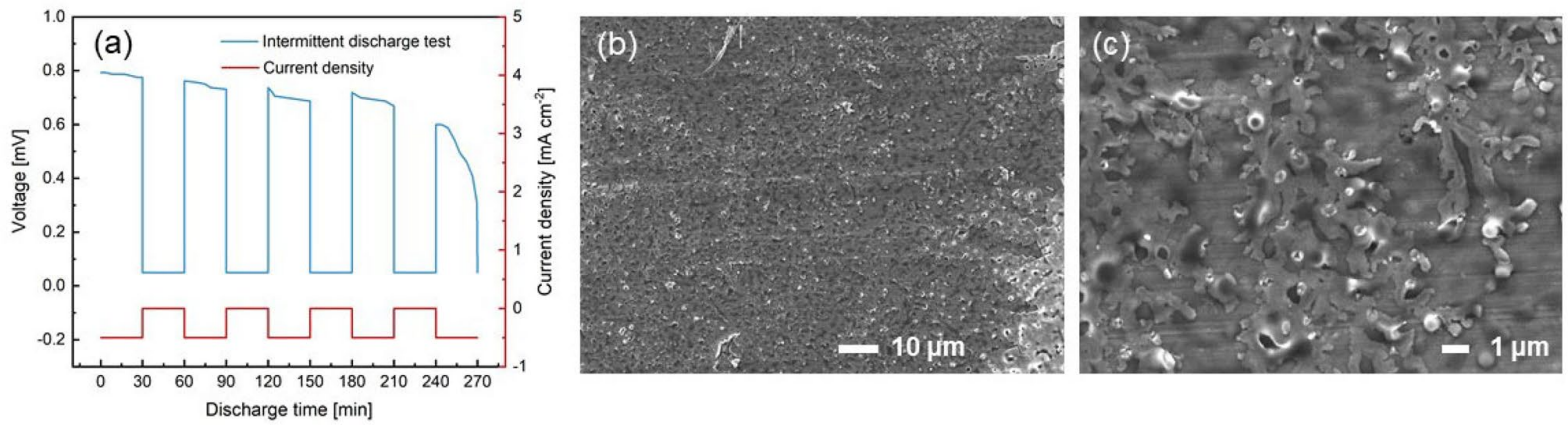
(**a**) The intermittent discharge profiles of the Al–air battery at 0.5 mA cm^−2^. (**b**,**c**) The morphology of the aluminum surface after discharge.

In order to further verify the stability of the electrolyte, this study takes an electrolyte with a thickness of 37.8 μm and a concentration of 1.8 mol as an example to assemble an Al–air battery and perform intermittent discharge. The current density is 0.5 mA cm^−2^, and the discharge was performed for 30 min at 30 min intervals. Figure 6a shows the intermittent discharge curves. As can be seen from the figure, the voltage of the interval discharge is still stable compared with the continuous constant current discharge. The discharge specific capacity is about 1506 mAh g^-1^, which is slightly higher than the specific capacity of the continuous discharge test. The morphology of the aluminum surface after discharge is shown in Fig. 6b,c. Many corrosion pits can be seen in the figures, which was the evidence of the local corrosion of Al contacted with NaCl gel electrolyte. The passivity morphology of the aluminum surface results from the surface hydroxide layer Al(OH)_3_. Similar results were also found in the study by Ma et al.^43^.

**Figure 7:**
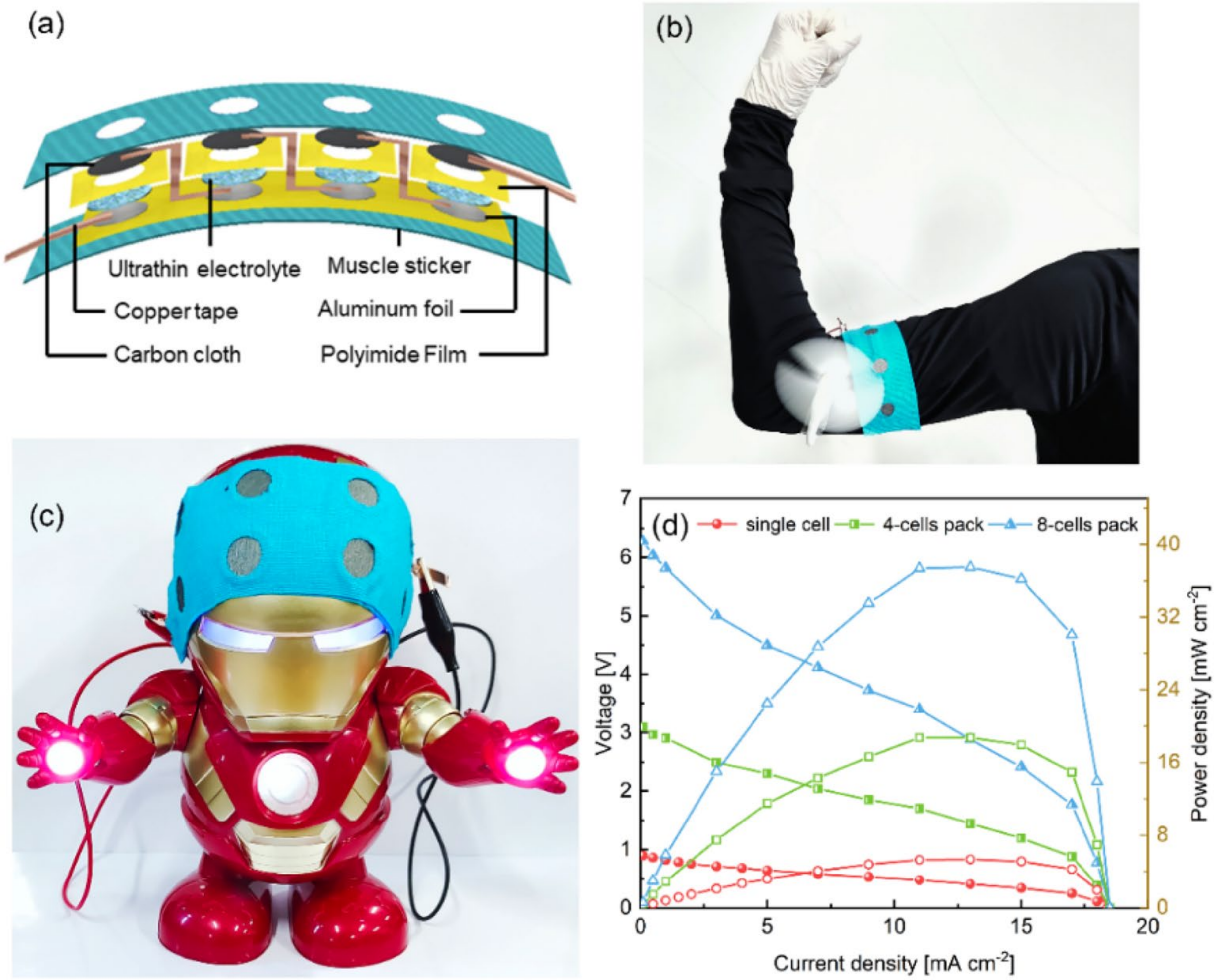
(**a**) Schematic diagram of the cell pack (Microsoft Office Hone and Student 2019-zh-cn https://www.microsoftstore.com.cn/software/office). The digital images of the cell pack with 4 (**b**,**c**) 8 single cells power a small fan and the Iron Man toy, respectively. (**d**) The polarization curves and power-density profiles of the cell pack and single cell.

In order to increase the output voltage and enhance the applicability of the Al–air batteries with ultrathin electrolytes, we connect single cells in series to form a wearable cell pack. The preparation process is shown in Fig. 7a. The cell pack consists of 4 single cells with a diameter of 1 cm. The Al anode, ultrathin electrolyte and air cathode are fixed on the muscle stickers. We cut out small holes in the muscle stickers according to the size of the single cell to ensure the supply of oxygen. Polyimide films are added as middle layers to fix the ultrathin electrolytes. The single cells are connected in series by copper tape, and the completed flexible Al–air cell pack is shown in Fig. 7b. The weight of the cell pack is about 115 g and the thickness is less than 1.3 mm. It can be attached to the surface of the skin or clothes according to actual needs. The cell pack uses the flexible ultrathin neutral electrolyte of this research, and there is no risk of electrolyte leakage. The cell pack is attached to the muscle sticker, and the Al anode is not direct contact with the skin. In addition, even if the Al anode is completely consumed under extreme conditions, the neutral electrolyte will not corrode the skin. A cell pack with 4 single cells can successfully power a small fan as show in Fig. 7b, and a cell pack with 8 single cells can power the Iron Man toy under bending state as shown in Fig. 7c. Figure 7d shows the comparison of the battery performance between the cell packs and the single battery. As shown in the figure, the peak power density of the cell pack with 8 single cells is 37.5 mW cm^-2^, which is about 7 times that of a single battery, and the packing efficiency is about 88%. Therefore, the cell pack can satisfy most low-power flexible wearable electronic products. The assembled cell pack can be directly attached to the skin or clothes. This fully demonstrates the good flexibility and applicability of the battery. The research results clearly indicate the potential application of the ultrathin flexible electrolyte in the field of flexible batteries.

## Conclusions

In summary, an ultrathin electrolyte for Al–air battery has been successfully prepared by aerosol jet printing. The research studied the influence of the thickness and concentration on conductance of the electrolyte in detail. The research results show that reducing the thickness and increasing the concentration of the electrolyte appropriately can effectively increase the conductance of the ultrathin electrolyte. The prepared ultrathin electrolyte has also been applied for flexible Al–air battery. Reducing the electrolyte thickness can indeed achieve better electrochemical performance of the battery, but it limits the specific capacity of the battery. The electrolyte concentration can be increased appropriately, and higher power density and specific capacity can be obtained. According to the influence of electrolyte thickness and concentration on battery performance, a suitable ultrathin electrolyte can be selected according to actual needs. In order to further improve the applicability of ultrathin electrolytes, we have prepared a cell pack assembled from single cells. The flexible cell pack can successfully drive small fans and Iron Man toys, which fully demonstrates its practicality. This study provides a new idea for the preparation of ultrathin electrolytes. The results are of great significance for the further commercial application of Al–air batteries in the field of wearable products.

## Methods

### Preparation of ultrathin electrolyte

**Figure 8:**
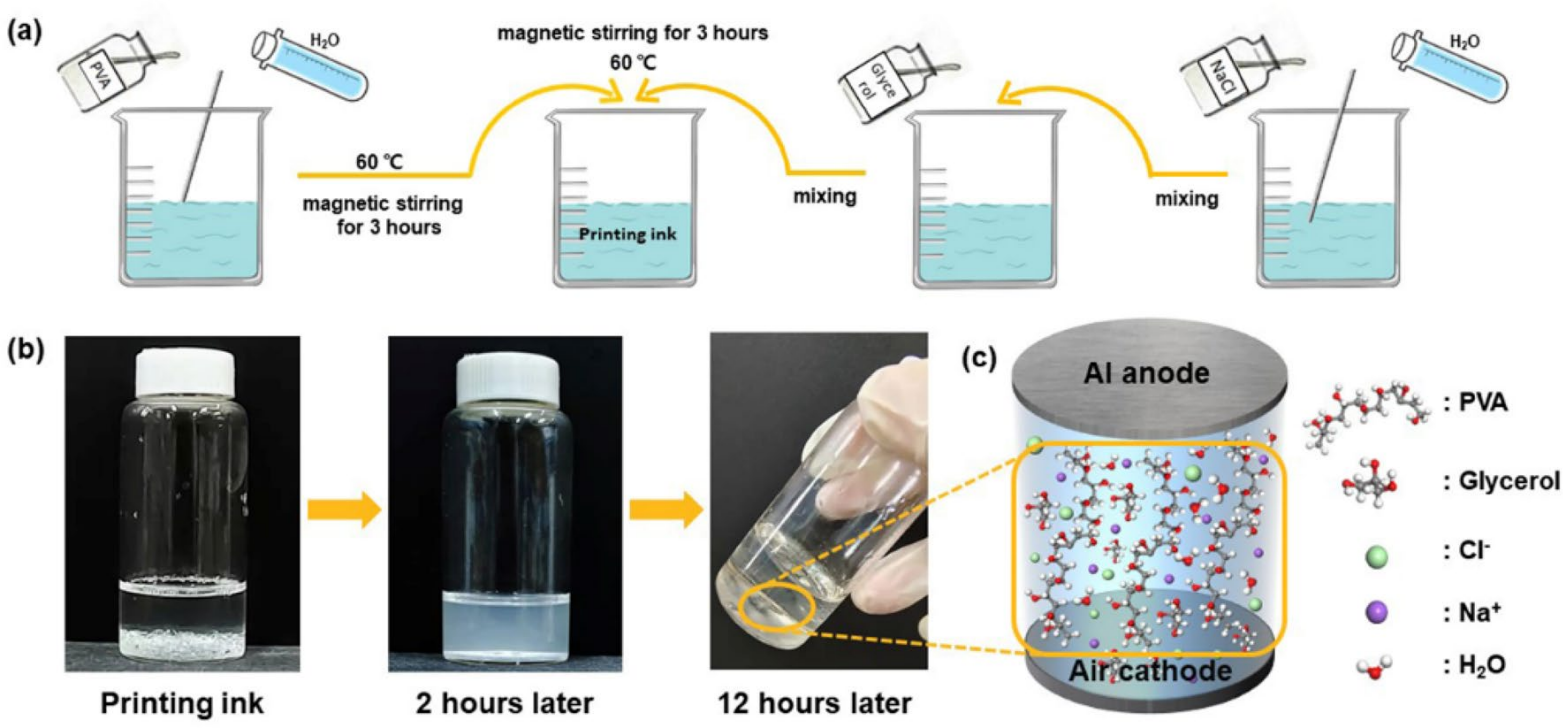
(**a**) The schematic illustration of synthesis of the printing ink. (**b**) Optical image of the printing ink changes over time. (**c**) Schematic diagram of Al–air battery structure and the electrolyte composition (Microsoft Office Hone and Student 2019-zh-cn https://www.microsoftstore.com.cn/software/office).

The previous study of Peng et al.^44^ conformed that PVA mixed with water, NaCl and glycerol could translate into the hydrogel through hydrogen bond cross-linking at room temperature. Based on the above research, this study improved the preparation process, adjusted the ratio of the ingredients, and formulated inks suitable for aerosol jet printing. The ink preparation is carried out under dry conditions. The schematic diagram of the preparation process is shown in Fig. 8a. Firstly, add 0.9 g NaCl to 5 ml pure water and stir until dissolved. Then add 0.5 ml glycerol to the above solution. 0.45 g PVA was mixed with 10 ml pure water and magnetically stirred at 60 ℃ for 3 h until the solution is uniform. Finally, the NaCl-glycerol and PVA solution should be mixed and magnetically stirred at 60 ℃ for 3 h. The viscosity of the final ink formulations is about 10 mPas, which is close to Newtonian behavior. Figure 8b shows the changes of the prepared printing ink over time at room temperature. After 2 h, the color of the solution became milky. After 12 h, the ink was in a gel state and with no fluidity. In our experiment, after the ink preparation is completed, it will immediately enter the printing process. After printing, it only needs to stand for 12 h to form a stable gel state, which can be used as an electrolyte for Al–air batteries as shown in Fig. 8c.

**Figure 9:**
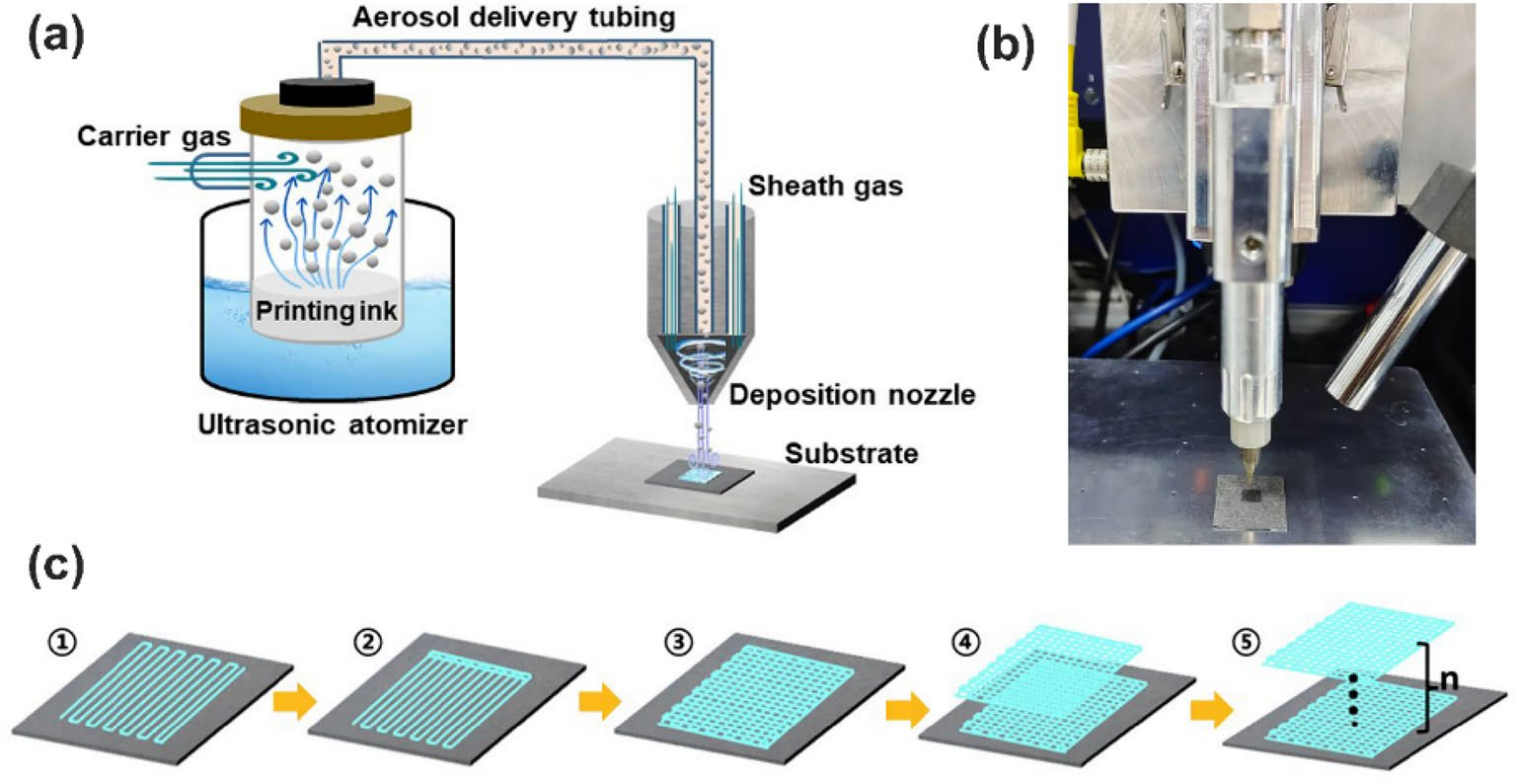
(**a**) Schematic diagram of aerosol jet printing (Microsoft Office Hone and Student 2019-zh-cn https://www.microsoftstore.com.cn/software/office). (**b**) Optical image of the aerosol printer in the process of printing electrolyte. (**c**) Printing process of the ultrathin electrolyte (Microsoft Office Hone and Student 2019-zh-cn https://www.microsoftstore.com.cn/software/office).

The aerosol jet printing was performed with an Optomec Aerosol Jet 200 printer. The schematic diagram of the printing mechanism is shown in Fig. 9a. The image of the electrolyte being printed is shown in Fig. 9b. The electrolyte printing was completed at a constant temperature of 25 ℃. The diameter of the nozzle is 200 μm, and the distance between the nozzle and the substrate is 10 cm. The ink flow rate is 10 mm s^-1^, and the ration of sheath gas to aerosol carrier gas is set to 3. The printing process is shown in Fig. 9c. Each layer is printed with a fold line to form a grid, and the next layer is printed with a misaligned grid to form an electrolyte membrane layer by layer. Every 10 layers of printing could form a complete electrolyte film. Electrolyte samples are prepared by printing 10–40 layers. After printing, all samples were dried at room temperature for 12 h.

### Physical characterizations

Surface morphology of the printed ultrathin electrolyte sample were characterized with SEM. The crystalline nature of the electrolyte sample was measured using XRD with a Cu kα radiation of 0.1541 nm as X-ray source.

### Electrochemical measurements

EIS test of the ultrathin electrolyte was conducted with a frequency range of 100 kHz–0.1 Hz and an amplitude of 10 mV using an electrochemical workstation. The Al–air battery is a sandwich structure, including a carbon cloth as an air cathode, the ultrathin electrolyte and an Al anode. The assembly flexible Al–air batteries were tested by constant current discharge with LAND test system.

## Data Availability

The datasets used and/or analysed during the current study available from the corresponding author Ying Yu on reasonable request.
